# Mechanistic Insights into the Action of Histamine-Functionalized PLA Nanoparticles Loaded with 5-Fluorouracil Against Gastric Cancer Cells *In Vitro*

**DOI:** 10.3390/molecules31142520

**Published:** 2026-07-20

**Authors:** Patrycja Jaroniek, Marek Brzeziński, Zuzanna Świniarska, Magdalena Chmiela, Weronika Gonciarz

**Affiliations:** 1 Department of Immunology and Infectious Biology, Institute of Microbiology, Biotechnology and Immunology, Faculty of Biology and Environmental Protection, University of Lodz, Banacha 12/16, 90-237 Lodz, Poland; patrycja.jaroniek@edu.uni.lodz.pl (P.J.); magdalena.chmiela@biol.uni.lodz.pl (M.C.); 2 Bio-Med-Chem Doctoral School of the University of Lodz and Lodz Institutes of the Polish Academy of Sciences, University of Lodz, Matejki 21/23, 90-237 Lodz, Poland; zuzanna.swinarska@cbmm.lodz.pl; 3 Centre of Molecular and Macromolecular Studies, Polish Academy of Sciences, Sienkiewicza 112, 90-363 Lodz, Poland; marek.brzezinski@cbmm.lodz.pl

**Keywords:** nanoparticles, gastric cancer, histamine

## Abstract

Gastric cancer is among the leading causes of cancer-related deaths worldwide. Modern treatment approaches include nanoparticles (NPs) designed to target cancer cells, which release a therapeutic cargo facilitating the inhibition of their expansion, thereby improving anti-tumor therapies. The success of NPs, created to deliver anticancer agents and biologically active compounds, may depend on selecting the way to target cancer cells. This study focused on examining the effects of NPs made of polylactic acid (PLA) with histamine (His) end groups and loaded with 5-fluorouracil (5-FU), a known anticancer drug (PLA-His-5-FU), on human gastric cancer AGS cells *in vitro.* The incubation of AGS cells with PLA-His-FU NPs resulted in diminished mitochondrial membrane potential and the induction of cell apoptosis, along with cell cycle arrest and the reduction of cell proliferation. Furthermore, the NPs tested provoked the secretion of pro-inflammatory cytokines tumor necrosis factor alpha (TNF-α) and interleukin (IL)-1β by AGS cells and induced the activation of the nuclear factor kappa B (NF-κB) signaling pathway in THP-1 blue monocytes, which indicates the ability to promote the development of a milieu for the infiltration and activation of immunocompetent cells. NPs did not increase intracellular adhesion molecule (ICAM-1) deposition on AGS cells, thus potentially preventing the distribution of cancer cells. In conclusion, PLA-His-5-FU NPs show promising anticancer activity for gastric cancer AGS cells *in vitro*, better than PLA-OH-5-FU, and can be used in further *in vivo* studies to confirm this activity.

## 1. Introduction

According to the International Agency for Research on Cancer (IARC), in 2022, gastric cancer (GC) was the fifth most common cancer, with over 960,000 new cases worldwide [1]. The most common risk factors include nicotine use, alcohol consumption, and poor diet, which can disrupt microbiome composition and lead to dysbiosis. Another predictive factor of gastric cancer is chronic infection with *Helicobacter pylori* (*H. pylori*). The virulence factors of these bacteria increase oxidative stress and DNA damage, epithelial cell damage, and the induction of pro-inflammatory responses, followed by hyperproliferation leading to malignant transformation [1,2,3,4,5]. Most patients are diagnosed in an advanced stage of cancer. Standard therapeutic procedures include tumor resection, chemotherapy, and immunotherapy [6,7]. Novel therapeutic strategies, primarily for patients after surgery or those diagnosed in the early stage of the disease, include multimodal or target-specific therapies. One of the most promising trends is the use of nanotechnology to design novel drug delivery systems (DDSs), which could facilitate the dose-controlled release of pharmacological agents and enhance drug effectiveness against cancer cells [8,9]. Our previous study showed that NPs based on PLA with His end groups and loaded with doxorubicin (DOX) diminished the expansion of gastric cancer cells *in vitro*, which is associated with increased oxidative stress, DNA damage, cell apoptosis, cell disintegration, and diminished cell proliferation [10].

Nowadays, chemotherapy with 5-FU is often mixed with epirubicin, cisplatin, or capecitabine [11]. However, the problems with the use of 5-FU include its low bioavailability, poor distribution and rapid degradation in vivo [12]. Nanoformulations may help overcome these drawbacks. The study conducted by Ugorji et al. in 2022 showed that non-ionic surfactant vesicles deliver an anticancer drug in a controlled manner, which results in increased drug accumulation [13]. Recently, various nanoformulations of AS1411 aptamer-modified liposomes have been tested on basal carcinoma cell lines to enhance drug cytotoxicity and cell penetration [14]. The study by Khodarahmi et al. [15] demonstrated that aptamer-conjugated calcium alginate and chitosan-coated liposomes containing 5-FU increased apoptosis and reduced resistance in colorectal adenocarcinoma HT-29 cells. Chen et al. [16] showed the effectiveness of cisplatin and 5-FU hydrogel in the treatment of gastric cancer. Several studies have developed 5-FU-loaded, chitosan-formulated nanoparticles, emphasizing their zeta potential in terms of carrier stability and drug bioavailability [17].

Although 5-FU-loaded PLA/PLGA-based nanocarriers have been widely investigated, including both passive polymeric systems and ligand-targeted formulations, the present formulation differs from these approaches by using His as an end group functionality incorporated into the PLA backbone [18,19]. Several previously reported 5-FU nanocarriers used targeting ligands such as folic acid, hyaluronic acid, or epidermal growth factor (EGF) to improve tumor–cell interaction and drug delivery efficiency [20,21,22]. By contrast, in the present formulation, His is not attached as a classical external targeting ligand but rather is introduced into the polymeric structure itself.

The rationale for histamine-functionalized PLA is based on the presence of the imidazole group, which may exhibit pH-sensitive protonation behavior and influence carrier–cell interactions, intracellular trafficking, and drug-delivery performance [23,24]. Moreover, NPs’ surface charge is an important determinant of cell interaction, uptake, intracellular localization, and cytotoxicity [25,26]. Therefore, His end group functionalization was expected to modify the physicochemical and biological properties of 5-FU-loaded PLA NPs.

In this study, PLA-His-5-FU was evaluated as an alternative end group-functionalized PLA nanocarrier and directly compared with non-functionalized PLA-5-FU under the same experimental conditions. The aim was to determine whether His functionalization of PLA can improve the physicochemical profile and biological activity of 5-FU-loaded NPs on gastric cancer AGS cells.

## 2. Results and Discussion

The *per os* administration of drugs for GC is promising; however, the acidic pH of gastric juice may affect drug stability and distribution [9,27,28]. Cancer cells may develop multi-drug resistance (MDR), which is associated with the tumor microenvironment, enhanced drug efflux, and alterations of apoptosis and the cell cycle, diminishing the cytotoxicity of the drug against cancer cells [29,30].

New nano-drug delivery systems (NDDSs) prevent drug degradation, facilitate the use of lower doses of anticancer drugs and help maintain the optimal drug concentration at malignant sites, as well as overcome MDR [9,29,30,31]. NDDSs based on PLA, possessing Food and Drug Administration (FDA) approval, enable the delivery of size-tailored NPs in controlled doses and facilitate the passive diffusion of NPs through malignant tissue [32,33,34,35,36,37]. Recently, the incorporation of additional substances that may enhance biological effects against cancer cells by increasing cell barrier permeability, improving drug pharmacokinetics, and exerting immunomodulatory properties has been proposed [16,35,38,39,40,41].

PLA has been used in several studies to develop NPs due to its high biocompatibility and structural properties facilitating the delivery of drugs, proteins, and nucleic acids [42,43]. The relatively small diameter of PLA-derived NPs facilitates the passive targeting of tumor cells [42,43]. Previously, PLA-based NPs were used for the encapsulation and delivery of 5-FU to solid Ehrlich carcinoma (SEC) [19], laryngeal cancer cells [44], and hepatocellular carcinoma [45]. In a previous study, we demonstrated that a combination of PLA functionalized with histamine residues enhances the efficacy of DOX-loaded NPs against gastric cancer cells and reduces the expression of tight junction proteins [10].

In this study, we used histamine-functionalized PLA loaded with 5-FU, the reference drug against gastric cancer [46], to improve the effectiveness of such a formulation against gastric cancer AGS cells *in vitro*. We verified this hypothesis by assessing mitochondrial potential, cell apoptosis, cell cycle, cell proliferating activity, and lipid peroxidation. Furthermore, several immune signatures have been assessed, including the secretion of proinflammatory cytokines TNF-α and IL-1β by AGS cells. THP-1 blue^®^ monocytes were used to assess the activation of the NF-κB signaling pathway. Additionally, ICAM-1 deposition on AGS cells has also been evaluated.

### 2.1. Characteristics of Nanoparticles

Selected initiators of L-lactide ring-opening polymerization were used to prepare PLA functionalized with hydroxyl and histamine end groups [10]. Nanoprecipitation was used to prepare blank PLA-OH NPs loaded with 5-FU (PLA-5-FU) and PLA-His NPs loaded with 5-FU (PLA-His-5-FU), and the composition of NPs was confirmed by FTIR (Figure S1). The FTIR spectra confirmed the expected chemical composition of the obtained PLA-based NPs. The spectra showed characteristic PLA bands, including ester carbonyl stretching, C–O/C–O–C stretching vibrations, and C–H stretching bands. In the spectra of His-functionalized NPs, additional bands corresponding to His-associated functional groups were observed, which supports successful PLA end group modification. In 5-FU-loaded formulations, spectral changes in the region corresponding to carbonyl, N–H/C–N and heterocyclic ring vibrations were consistent with the presence of 5-FU in the matrix. These results confirm the formation of PLA-5-FU or PLA-His-5-FU formulations. The size of the PLA-OH NPs was 103 nm, whereas the size of the PLA-His NPs was 147 nm (Figure S2, Table S1). The encapsulation of 5-FU increased the NPs’ size: PLA-5-FU to 121 nm and PLA-His-5-FU to 150 nm. Moreover, an unexpected but reproducible change in zeta potential was observed after 5-FU loading into PLA-His NPs (Figure S2, Table S1). Blank PLA-His NPs showed a negative zeta potential of −18.5 mV, whereas PLA-His-5-FU NPs showed a positive zeta potential of +5.2 mV. By contrast, 5-FU loading into PLA-OH NPs resulted only in a moderate shift from −6.8 mV to −10 mV. Because 5-FU is weakly acidic and not cationic, this reversal should not be interpreted as a direct positive charge contribution from 5-FU itself.

Zeta potential reflects the electrokinetic potential at the slipping plane and is influenced not only by the formal charge of individual molecules but also by the surface organization, polymer chain orientation, exposure or shielding of ionizable groups, hydration layer, counterion distribution, and drug-induced reorganization of the nanoparticle interface [25,47]. Therefore, the positive zeta potential observed for PLA-His-5-FU is most likely related to a formulation-specific surface rearrangement after 5-FU incorporation into the His-functionalized PLA matrix.

This interpretation is supported by physicochemical data. PLA-5-FU and PLA-His-5-FU showed comparable encapsulation efficiency, but the release of 5-FU from PLA-His-5-FU was slower than that from PLA-5-FU, which suggests that His residues influence drug–carrier interactions. The release data fit the Korsmeyer–Peppas model for both formulations; however, PLA-His-5-FU showed a lower kinetic constant, which further supports a distinct interaction between 5-FU and the histamine-functionalized polymer matrix (Table S2). These observations are consistent with the hypothesis that 5-FU incorporation may alter the exposure of His/imidazole-related groups and shield negatively contributing surface groups, thereby shifting the apparent zeta potential toward positive values.

The possible contribution of His residues is biologically plausible because imidazole-containing structures, including histidine/His-related moieties, can exhibit pH-dependent protonation behavior and are widely used in pH-responsive delivery systems [23,24,48]. Moreover, surface charge is known to affect nanoparticle–cell interactions, cellular uptake, intracellular localization, and cytotoxicity [25,26]. Thus, the positive zeta potential of PLA-His-5-FU may contribute to a stronger interaction with AGS cells, but it should not be considered the sole or definitively proven mechanism of enhanced uptake or cytotoxicity.

The present study does not directly prove the molecular basis of this zeta-potential reversal. Therefore, mechanistic statements related to surface charge, uptake, and cytotoxicity have been revised and are now presented as hypotheses rather than definitive conclusions. Further studies should include pH-dependent zeta potential measurements, surface-sensitive chemical analysis such as XPS or ToF-SIMS, NMR or ITC studies of 5-FU–His interactions, and zeta potential-matched control NPs to determine whether the observed biological activity is directly caused by surface charge or by other effects of His functionalization.

Concerning these data, the developed NPs are appropriate for delivering 5-FU to gastric cancer cells and internalization through pinocytosis [49]. The release of 5-FU from NPs was examined in PBS at 37 °C (Figure S3). Obtained NPs can be stored for one month without aggregation (Figure S3), and their redispersion in PBS does not influence the NPs’ size, which indicates their desirable colloidal stability. SEM analysis showed well-defined spherical particles formed by nanoprecipitation, consistent with the findings of Jose et al. [50] and Tural and Acartürk [51].

### 2.2. The In Vitro Release of 5-FU and NP Cell Uptake

The encapsulation efficiency (EE) of 5-FU was similar for both formulations, reaching 34.8% for PLA-5-FU NPs and 34.6% for PLA-His-5-FU NPs, indicating that His end group functionalization did not improve 5-FU encapsulation efficiency (Figure S3, Table S1). The release of 5-FU from the NPs was tested in PBS at 37 °C. During the first 6 h, both formulations showed a burst release, probably due to the fraction of 5-FU located near or weakly bound to the NPs’ surface. This was followed by a slower, sustained release over 72 h, likely caused by the diffusion of 5-FU from the polymer matrix. The total 5-FU released from PLA-His-5-FU NPs was lower than that from PLA-5-FU NPs, at 28% and 39%, respectively. This suggests that His end groups did not increase drug loading but rather affected the release of 5-FU. Kinetic analysis confirmed this, with the data fitting the Korsmeyer–Peppas model, indicating a diffusion-controlled release (Table S2). The lower total release and slower rate for PLA-His-5-FU NPs suggest stronger or more specific interactions between 5-FU and the His-functionalized PLA matrix. This could be due to imidazole-containing His residues, which may engage in pH-dependent interactions and influence the NPs’ interface organization [23,24]. Together with the change in zeta potential, these observations show that His end group functionalization alters the physicochemical properties of 5-FU-loaded PLA NPs. Nevertheless, the improved biological activity of PLA-His-5-FU compared with PLA-5-FU should be interpreted with caution, because this study did not directly establish whether it results from changes in surface charge, drug release, cellular uptake, intracellular trafficking, or a combination of these factors [25,26]. The limitation of this study is that PLA-His-5-FU was not directly compared with well-known targeting ligands or charge-altered NPs systems such as folate-, hyaluronic acid-, peptide-, antibody-, aptamer-, EGF-, or cationic polymer-modified nanocarriers. Consequently, we do not claim that PLA-His-5-FU outperforms these systems. Instead, our findings position PLA-His-5-FU as an alternative end group-functionalized PLA-based formulation that shows increased activity over non-functionalized PLA-5-FU in the gastric cancer AGS cell model. Future studies should quantify cellular uptake, measure intracellular 5-FU, use controls with matched zeta potential, perform receptor blocking or competitive inhibition tests, investigate endosomal trafficking, and compare with other ligand or charge modification strategies.

**Figure 1:**
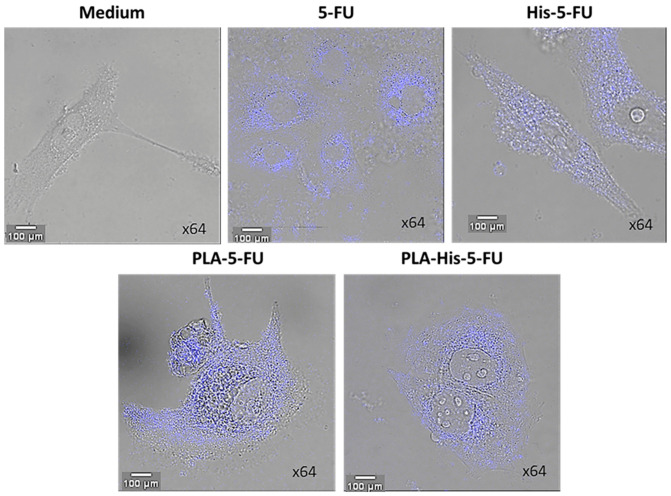
**Confocal microscopy images showing cellular association/internalization of 5-FU or His-5-FU.** The effect was assessed qualitatively based on measurement of native blue fluorescence of 5-FU/His-5-FU at 405 nm excitation with violet laser, and emission was collected at 450 nm/430–480 nm. No external fluorescent dye was used. The images were captured using a Leica SP-8 confocal microscope (Leica Microsystems GmbH, Wetzlar, Germany) at a magnification of 64×.

Confocal microscopy images demonstrated the cellular uptake of the tested NPs (PLA-5-FU and PLA-His-5-FU) and 5-FU or His-5-FU after 24 h of incubation with AGS cells (Figure 1). The penetration assay showed that NPs can bypass the cell membrane barrier of gastric cancer cells. The well penetration of PLA-His-5-FU NPs may partly result from a positive zeta potential (+5.2 mV) (Table S1). Consequently, the subsequent sections of this study were designed to explore the anticancer effects of PLA-5-FU vs. PLA-His-5-FU NPs.

### 2.3. NP Cytotoxicity Pathways Against AGS Cells

**Figure 2:**
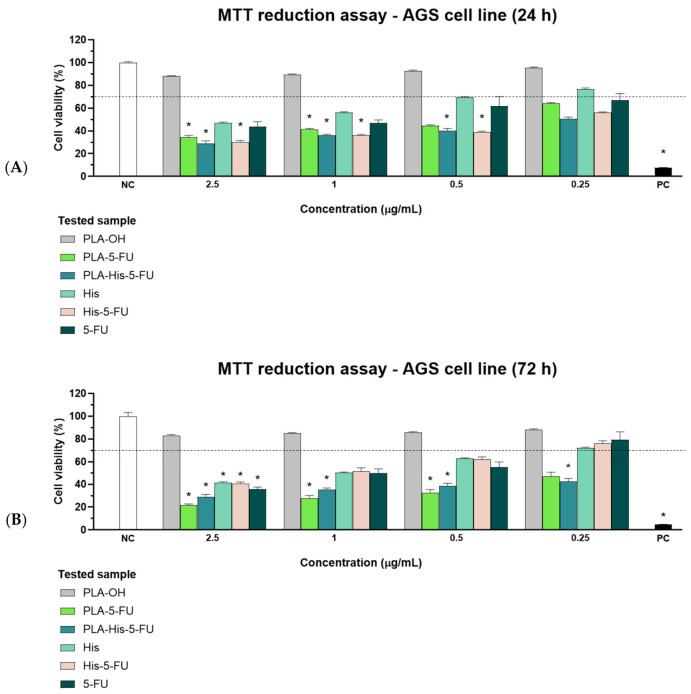
**The cytotoxicity of PLA-based nanoparticles (NPs) against human gastric cancer AGS cells.** The dotted line indicates 70% viability, demonstrating the lack of cytotoxic effect. Median values after (**A**) 24 h and (**B**) 72 h are shown. * cells in medium vs. cells exposed to tested NPs.

In the first step, the *in vitro* cytotoxicity of the tested NPs against the reference L929 fibroblasts was assessed according to ISO 10993-5 [52] (Figure S4). Next, the influence of 5-FU, His, and His-5-FU alone, as well as the synthesized NPs PLA-OH, PLA-5-FU, PLA-His, and PLA-His-5-FU on AGS cells was assessed (Figure 2). NPs were considered biocompatible when cell viability was not lower than 70%. The NPs tested—PLA-OH, PLA-5-FU, PLA-His, and PLA-His-5-FU—were not cytotoxic against the L929 murine fibroblasts after 24 h of incubation at a 1 µg/mL concentration. Only soluble His, but not His-5-FU, diminished the viability of L929 cells below 70% at 0.5 µg/mL (Figure S4). It has been shown that soluble His may affect cell viability, potentially due to its ability to influence cell-to-cell integrity and cell membrane permeability [53,54]. However, His cytotoxicity was lower when incorporated in the NPs’ matrix or in complex with 5-FU. In AGS cell cultures, PLA-5-FU NPs diminished cell viability to 65%, while PLA-His-5-FU NPs diminished it to 50% at 0.25 µg/mL. The effect of PLA-His-5-FU was stronger than that of PLA-5-FU after 24 h of cell exposure, potentially due to the zeta potential (+5 mV) of PLA-His-5-FU. Histamine contains an imidazole ring with a pKa around 6.0. In the neutral environment of the blood (pH 7.4), it is uncharged. However, in the acidic environment of the endosome (pH~5.0–6.0), the imidazole group becomes protonated (Table S1). Comparing the cytotoxic concentration of NPs tested against non-cancer L929 cells (1 µg/mL) vs. gastric cancer AGS cells (0.25 µg/mL), it seems that AGS cells were more sensitive to these NP formulations than non-cancer cells, which may be a promising feature of these NPs concerning potentially lesser side effects. Zamora-Mora et al. [55], in an *in vitro* cytotoxicity study of chitosan NPs loaded with 5-FU, showed the stronger cytotoxic activity of these NPs against normal cells than against human glioblastoma cancer cells. This effect may be associated with cancer cells’ resistance to 5-FU or with the inactivation of enzymes involved in cell proliferation [56].

To clarify the dose-dependent effect of the tested compounds and formulations, dose–response curves were generated from the MTT viability data, and apparent IC_50_ values were calculated for all treatment groups (Table 1). PLA-OH did not reach IC_50_ within the tested concentration range, and its IC_50_ was, therefore, reported as >2.5 µg/mL at both 24 h and 72 h. For PLA-5-FU, the apparent IC_50_ was approximately 0.40 µg/mL at 24 h and <0.25 µg/mL at 72 h. For PLA-His-5-FU, the apparent IC_50_ was approximately 0.25 µg/mL at 24 h and <0.25 µg/mL at 72 h. The apparent IC_50_ values for soluble His were approximately 1.74 µg/mL at 24 h and 0.98 µg/mL at 72 h, while for His-5-FU, they were approximately 0.31 µg/mL at 24 h and 1.07 µg/mL at 72 h. Soluble 5-FU showed apparent IC_50_ values of approximately 0.84 µg/mL at 24 h and 0.90 µg/mL at 72 h. These results confirm that the cytotoxic effect was dose-dependent and that the His-functionalized 5-FU-loaded NPs showed the strongest activity among the tested 5-FU-containing formulations. PLA-His-5-FU reduced AGS cell viability even at the lowest tested concentration and showed a lower apparent IC_50_ than PLA-5-FU after 24 h, which indicates the enhanced biological activity of the His-functionalized formulation.

The lowest tested concentration, 0.25 µg/mL, was used to identify the first detectable cytotoxic response in the MTT screening. However, it was not used as a working concentration for the downstream mechanistic assays. Qualitative uptake/cell imaging was performed at 0.5 µg/mL for 24 h, whereas all subsequent mechanistic assays were conducted at 1 µg/mL. The concentration of 1 µg/mL was selected because it represented a biologically active and technically suitable mechanistic working concentration. At this concentration, the tested PLA-based NPs showed cytocompatibility with reference L929 fibroblasts according to the ISO 10993-5 criterion, while gastric cancer AGS cells were sensitive to the studied formulations. Therefore, the mechanistic data obtained at 1 µg/mL should be interpreted as explaining the cellular response to PLA-His-5-FU at the selected mechanistic working concentration. These findings complement the full dose–response cytotoxicity analysis and support the conclusion that His-functionalized PLA NPs enhance the biological activity of 5-FU against gastric cancer AGS cells.

**Figure 3:**
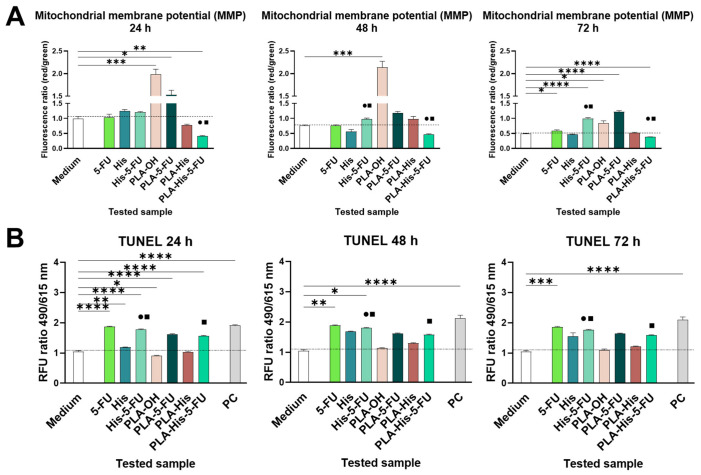
**Mitochondrial membrane potential and cell apoptosis in response to PLA-composed NPs**. Mitochondrial membrane potential (**A**) and cell apoptosis (**B**) of AGS cells were assessed after 24, 48, and 72 h of cell exposure to tested nanoparticles (NPs) or soluble components (1 µg/mL). The experiment was performed in triplicate for each experimental variant. * cells in medium vs. cells exposed to tested compounds. • His vs. His-5-FU or PLA-5-FU vs. PLA-His-5-FU. ▪ 5-Flu vs. His-5-FU or PLA-His vs. PLA-His-5-FU. ** *p* < 0.01, *** *p* < 0.001, and **** *p* < 0.0001.

A high mitochondrial membrane potential (MMP) in cancer cells increases metabolic activity, which is associated with tumor growth and invasiveness. The study by Begum and Shen et al. [57] showed that reduced MMP levels lead to cell apoptosis. The incubation of AGS cells with PLA-OH or PLA-5-FU resulted in increased MMP after 24 h, 48 h, and 72 h of incubation. By comparison, in cell cultures treated with PLA-His-5-FU, MMP decreased over the course of the culture (24–72 h), and the difference was statistically significant compared to the control (Figure 3A).

The fundamental role of novel therapeutics is to promote cancer cell apoptosis and limit cell proliferation [58,59]. In this study, increased cell apoptosis statistically significantly increased after 24 h of cell exposure to PLA-5-FU and PLA-His-5-FU, and this effect persisted for 72 h, although without statistical significance compared to the control (Figure 3B). Increased cell apoptosis was also observed in cell cultures containing soluble 5-FU or His-5-FU, while mitochondrial membrane potential remained at control levels, which suggests differences in the mechanisms of apoptosis initiation.

It has also been shown that the NPs tested—PLA-OH, PLA-5-FU, and PLA-His-5-FU—did not increase lipid peroxidation in AGS cells (Figure S5). However, increased oxidative stress in the cancer milieu may be beneficial in terms of therapeutic effects regarding DNA damage. It has been shown that therapeutics that modulate lipid peroxidation can enhance the overall cytotoxic effect of anticancer treatment [60]. The study by Xu et al. [61] revealed that elevated lipid peroxidation may mitigate the loss of sensitivity of gastric cancer cells to chemotherapeutics.

**Figure 4:**
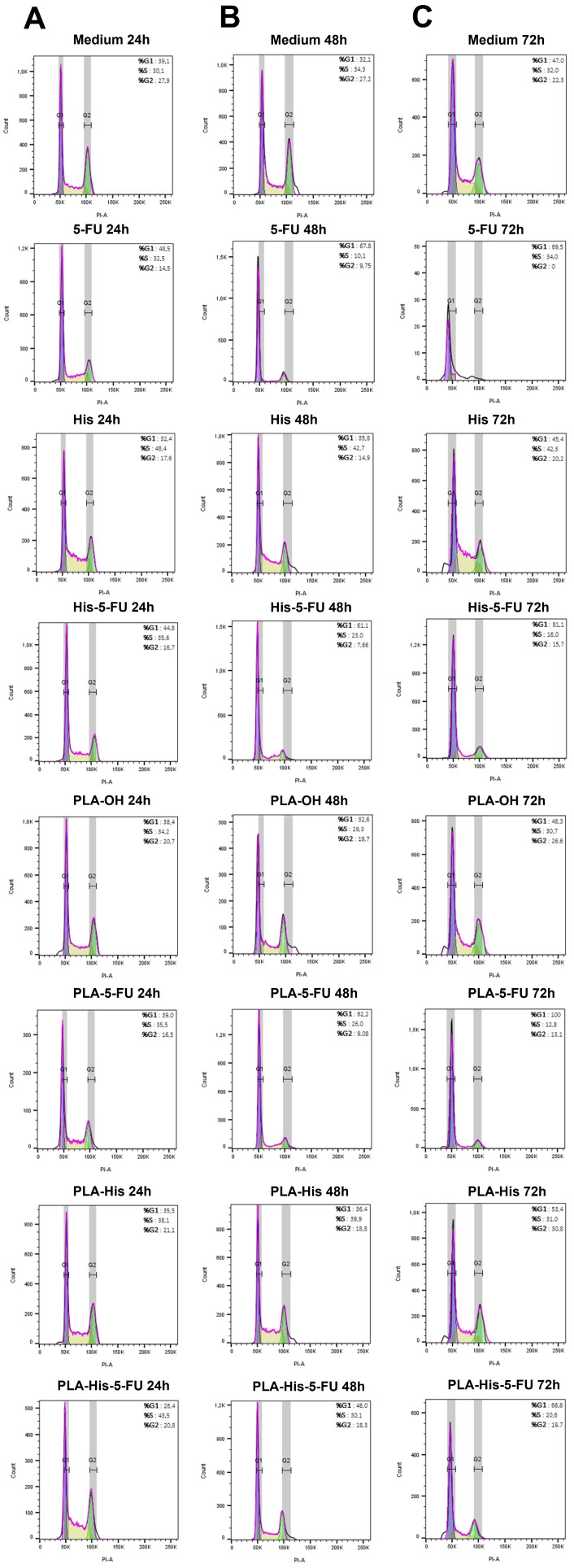
**Representative histograms of cell cycle phase distribution in human gastric cancer AGS cells.** The cell cycle phase distribution was assessed after cell exposure to nanoparticles (NPs) composed of polylactic acid (PLA-OH) functionalized with histamine (PLA-His) or such NPs loaded with 5-fluorouracil (PLA-His-5-FU), or soluble components (1 µg/mL). The DNA content was measured after 24 h (**A**), 48 h (**B**), and 72 h (**C**).

**Figure 5:**
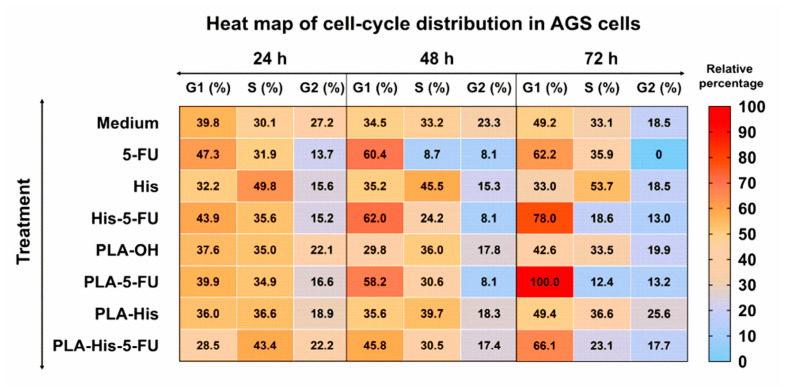
**Cell cycle phase distribution of human gastric cancer AGS cells after cell exposure to PLA-composed nanoparticles (NPs).** The percentage of cells distributed in G0/G1, S, and G2 phases was assessed by flow cytometry and analyzed in FlowJo software (https://flowjo.com/). Values are presented as means.

The increased cytotoxicity of PLA-His-5-FU NPs against AGS cells may be related to cell cycle arrest and the inhibition of cell proliferation. As shown in Figure 4 and Figure 5, PLA-composed NPs influenced a cell cycle phase distribution in AGS cells. There was a significant increase in AGS cells in the S phase after cell exposure for 24 h to PLA-His-5-FU (43.4%) or His (49.8%) compared to control cells (30.1%). At the same time, the percentage of AGS cells in the G2 phase was significantly decreased in the cell cultures containing PLA-His-5-FU (22.2%) or His (15.6%, non-significant tendency) vs the control cell culture (27.2%), which suggests S phase arrest. Furthermore, there was a significantly lower percentage of AGS cells in the G2 phase when exposed to 5-FU (13.7%), which suggests that 5-FU may influence cell cycle progression from the S phase to the G2/M phase. Notably, upon exposure of the cells to 5-FU, PLA-5-FU, or PLA-His-5-FU for 48 or 72 h, the percentage of AGS cells in the G1 phase showed a tendency to increase, with a simultaneous reduction in the S and G2M phases, which suggests the inhibition of cell cycle progression in the G0/G1 phase.

**Figure 6:**
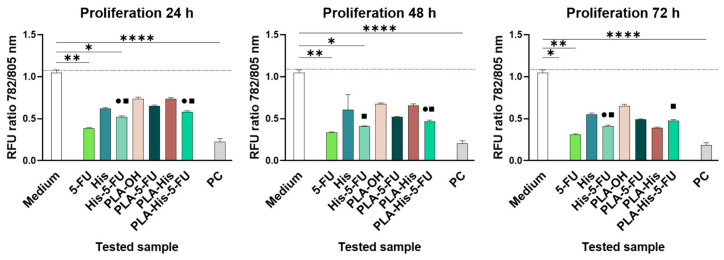
**Proliferation of human gastric cancer AGS cells after stimulation with PLA-composed nanoparticles.** The cell proliferating activity was assessed fluorometrically after 24 h, 48 h, or 72 h of cell exposure to nanoparticles (NPs) composed of polylactic acid (PLA-OH) functionalized with histamine (PLA-His) loaded with 5-fluorouracil (5-FU) or soluble compounds (1 µg/mL). * cells in medium alone vs. cells exposed to tested compounds. • His vs. His-5-FU or PLA-5-FU vs. PLA-His-5-FU. ▪ 5-Flu vs. His-5-FU or PLA-His vs. PLA-His-5-FU. ** *p* < 0.01, and **** *p* < 0.0001.

Moreover, 5-FU, PLA-His-5-FU, His, and 5-FU were associated with diminished cell proliferation, as determined using a fluorescent DNA probe (Figure 6). It has been shown that the coincubation of AGS cells with His or 5-FU alone, or with PLA-5-FU, PLA-His, and particularly PLA-His-5-FU NPs, resulted in diminished cell expansion compared to such spontaneous activity in culture medium alone (Figure 6).

### 2.4. Induction of Immune Signatures by PLA-Composed NPs

**Figure 7:**
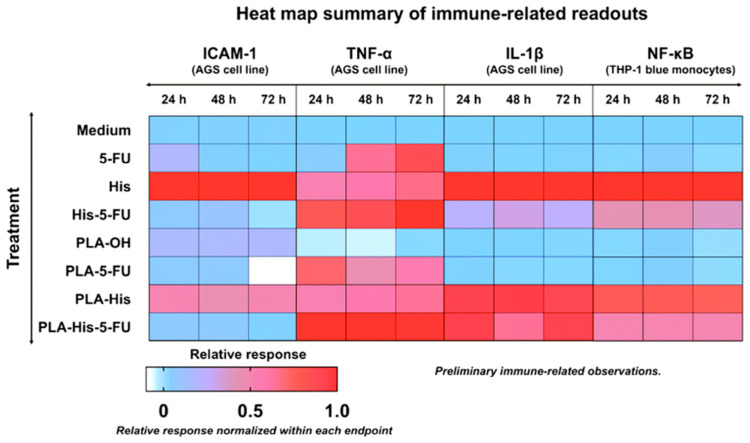
**Heat map of immune-related readouts (TNF-α, ICAM-1, IL-1β, NF-κB).**

The influence of PLA-derived NPs loaded with 5-FU or free 5-FU, His, and their combination on selected immune signatures is shown in Figure 7. PLA-5-FU or PLA-His-5-FU NPs did not modulate ICAM-1 deposition on AGS cells during cell culture. Only His alone significantly increased ICAM-1 deposition on these cells, while PLA-His showed a similar tendency. ICAM-1 upregulation in gastric cancer cells can promote vascular diapedesis. In this context, the lack of enhanced ICAM-1 deposition by PLA-His-5-FU may be beneficial. On the other hand, these changes may facilitate the recognition of cancer cells by immunocompetent cells; however, this interpretation requires further validation in tumor–immune co-culture models [62,63].

This study demonstrated that NPs functionalized with His and loaded with 5-FU can promote the development of a favorable environment for the activation of immunocompetent cells by stimulating the production of pro-inflammatory cytokines by AGS cells. These cells responded by significantly enhancing TNF-α production when exposed to PLA-His-5-FU or His-5-FU (Figure 7). Regarding IL-1β, the increased level of this cytokine was observed in cell cultures exposed to PLA-His-5-FU, PLA-His, His-5-FU, and His but not 5-FU, which suggests the dominant role of His (Figure 7). Similarly, PLA-His-5-FU and PLA-His, His-5-FU or His activated the NF-κB signaling pathway in THP-1 blue monocytes (Figure 7). These findings show that newly designed PLA-His-5-FU NPs can create a pro-inflammatory milieu due to the activation of cancer cells to cytokine secretion and the activation of monocytes, which may have a direct anticancer activity or may be involved in inducing the development of an adaptive immune response as antigen-presenting cells. The complex development procedure of NPs may not exclude the endotoxin content in the final formulation. Therefore, the immunomodulatory effects of the studied NPs should be interpreted with caution. Future studies should include LAL-based endotoxin quantification of all NP formulations and soluble components, together with spike-recovery controls to exclude NPs’ interference with endotoxin detection. In addition, endotoxin-neutralization experiments using polymyxin B would be required to confirm whether the observed cytokine and NF-κB responses are specifically related to His-enriched formulations.

The present study showed that PLA-His-5-FU NPs exerted stronger biological effects on gastric cancer AGS cells than free 5-FU, His-5-FU, PLA-5-FU, or PLA-His alone. PLA-His-5-FU reduced cell viability, decreased mitochondrial membrane potential, promoted DNA fragmentation, affected cell cycle distribution, and reduced cell proliferation. These findings suggest that His end group functionalization may enhance the biological activity of 5-FU-loaded PLA nanocarriers.

In immune assays, both PLA-His-5-FU and His-5-FU increased TNF-α levels. Additionally, PLA-His-5-FU, PLA-His, His-5-FU, and His elevated IL-1β and stimulated NF-κB activation. These findings imply that His formulations might induce a pro-inflammatory environment, supporting immune cell activation. However, without detailed drug interaction studies, these observed effects likely represent the combined or amplified influence of His and 5-FU rather than true synergy.

**Figure 8:**
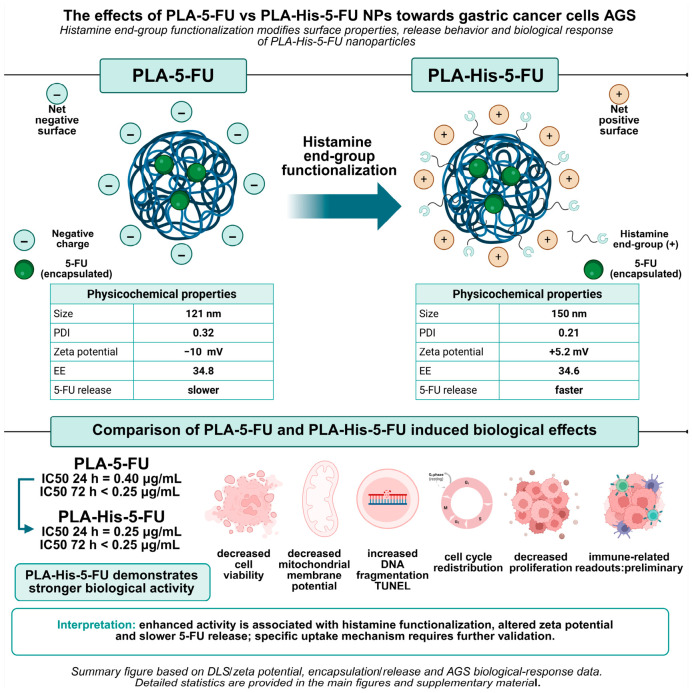
**Integrated summary of the principal differences between PLA-5-FU and PLA-His-5-FU nanoparticles.** The figure summarizes the main physicochemical and biological features distinguishing non-functionalized PLA-5-FU from histamine-functionalized PLA-His-5-FU. The comparison includes nanoparticle size, zeta potential, 5-FU release behavior, cytotoxic response against AGS cells, mitochondrial membrane potential, DNA fragmentation, cell cycle redistribution, proliferation, and selected immune-related readouts. The scheme highlights that histamine end group functionalization modifies the physicochemical properties of the 5-FU-loaded PLA system and is associated with enhanced biological activity against gastric AGS cancer cells *in vitro*. Created in BioRender. Mikolajczyk-Chmiela, M. (2026) https://BioRender.com/obhh2ui.

Time-dependent responses at 24, 48, and 72 h were presented as line graphs to illustrate temporal changes in mitochondrial membrane potential, DNA fragmentation, proliferation, lipid peroxidation, cytokine secretion, ICAM-1 deposition, and NF-κB activation. In addition, cell cycle distribution and immune-related endpoints were summarized in heatmaps to facilitate easier comparison of global response patterns across treatment groups. This visualization outlines the primary comparison between PLA-5-FU and PLA-His-5-FU, summarizing key physicochemical and biological insights in a figure that underscores how His end group functionalization influences 5-FU-loaded PLA nanoparticles (Figure 8).

An important limitation of the present study is that the complex NP preparation procedure does not exclude the possibility of endotoxin contamination in the final formulations. Therefore, the immunomodulatory effects of the studied NPs should be interpreted with caution. Future studies should include LAL-based endotoxin quantification of all NP formulations and soluble components, together with spike-recovery controls to exclude NPs’ interference with endotoxin detection. In addition, endotoxin-neutralization experiments with polymyxin B would be required to confirm whether the observed cytokine and NF-κB responses are specifically associated with His-enriched formulations.

## 3. Materials and Methods

### 3.1. Materials

Tin (II) octoate was sourced from Sigma-Aldrich (St. Louis, MO, USA), distilled using calcium hydride, and kept under reduced pressure. L, L–LA (99%, Purac, Gorinchem, The Netherlands) was crystallized from dried 2-propanol, sublimated, and likewise stored under reduced pressure. Histamine (Sigma-Aldrich, 97%) underwent purification through sublimation and was maintained under reduced pressure. THF (Chempur, Piekary Śląskie, Poland, 99%) was processed over KOH pellets, filtered, refluxed over sodium, distilled, and condensed into an ampoule containing liquid Na/K alloy. Methanol was obtained from Chempur (Poland), distilled over sodium, and stored under vacuum with 4 Å molecular sieves. 5-fluorouracil (5-FU), poly (ethylene glycol) methyl ether-block-poly(D,L-lactide) (PEGME-b-PDLLA), MTT [3-(4,5-dimethylthiazol-2-yl) 2,5-diphenyltetrazolium bromide], and phosphate-buffered saline (PBS) at pH 7.4 were also acquired from Sigma-Aldrich (Sigma-Aldrich) and used without further purification.

### 3.2. Methods

#### 3.2.1. Characterization of Methodology and Equipment

We acquired 1H NMR spectra in chloroform-d1 using a Bruker DRX500 spectrometer (Bruker, Bremen, Germany) functioning at 500 MHz. The polymers’ number-average molecular weights (Mns) were determined through size-exclusion chromatography (SEC) on an Agilent Pump 1100 Series (preceded by an Agilent G1379A Degasser, Santa Clara, CA, USA) utilizing two PLGel 5 μm MIXED-C columns (Wyatt Technology, Drenbach, Germany). Dichloromethane was used as the eluent at a flow rate of 0.8 mL min^−1^ at room temperature. Fourier-transform infrared spectroscopy (FTIR) measurements were conducted with a Nicolet 6700 spectrometer featuring a deuterated triglycine sulfate (DGTS) detector (Thermo Scientific, Madison, WI, USA). Attenuated total reflectance (ATR) was employed for these IR measurements, with the spectra obtained by averaging 64 scans at a resolution of 2 cm^−1^.

#### 3.2.2. Preparation of NPs by Nanoprecipitation

The initial step in creating NPs involved the synthesis of polylactic acid (PLA) with specific end groups [10]. The PLA (Merck KGaA, Darmstadt, Germany) that included histamine (His) was synthesized either in the presence of a catalyst (stannous octoate) or by using His as both an initiator and a polymerization catalyst. Next, we applied the nanoprecipitation method [19] to prepare either blank NPs or those loaded with 5-FU from the synthesized PLA with His end groups. We chose nanoprecipitation techniques because NPs loaded with 5-FU or possessing His end groups exhibited greater cytotoxic effects compared to those produced via the microfluidic method [10].

#### 3.2.3. Encapsulation Efficiency and In Vitro Drug Release

To determine the release profile of 5-FU from NPs, a defined volume of the sample (1 mL of nanoparticle suspension) was transferred into a Float-A-Lyzer^®^ device (Spectra/Por^®^ (Waltham, WA, USA), 3.5–5 kDa). The release experiment was initiated by immersing the cassette in 5 mL of phosphate buffer saline (PBS, 0.1 M, pH 7.4). At predetermined time intervals, 1 mL of the release medium was withdrawn for measurement and then returned to the release medium. The entire release medium was maintained at 37 °C. The amount of released FU was determined spectrophotometrically using a Thermo Scientific Evolution 220 UV-Vis spectrophotometer (Waltham, MA, USA) at a wavelength of 265 nm, based on a previously prepared calibration curve for 5-FU. Encapsulation efficiency (EE, %) was calculated as EE (%) = [(W total 5-FU − W free 5-FU)/W total 5-FU] × 100, where W total 5-FU is the initial amount of 5-FU used for NP preparation and W free 5-FU is the amount of non-encapsulated 5-FU detected in the supernatant after NP separation. The experiments were repeated three times.

#### 3.2.4. Cell Culture Conditions

The L929 mouse fibroblasts (purchased from LGC Standards, Middlesex, UK) and human gastric cancer AGS cells (purchased from American Type Culture Collection; ATCC^®^ CRL-1739™, Manassas, VA, USA) were used for in vitro cytotoxicity testing. The cells were maintained under standard conditions (37 °C, 5% CO_2_) in 25 cm^2^ tissue culture flasks in RPMI-1640 medium (Sigma-Aldrich, St. Louis, MO, USA) supplemented with 10% fetal bovine serum (FBS) (Cytogen, Lodz, Poland) and antibiotics: 100 U/mL penicillin and 100 µg/mL streptomycin (Polfa Tarchomin S.A., Warszawa, Poland), as previously described [64]. Prior to the experiments, cell viability was assessed by trypan blue exclusion and remained in the range of 93–95%.

Different variants of AGS cell cultures were used: cells in culture medium alone—negative control (NC) or cells stimulated for 24 h only with 5-FU, His, and His-5-FU or NPs obtained with a catalyst by nanoprecipitation method: empty PLA-OH, PLA-His, PLA-OH loaded with 5-FU (PLA-5-FU), or PLA-His loaded with 5-FU (PLA-His-5-FU). All stimulants were used at a concentration of 1 μg/mL, while the reference anticancer drug DOX was a positive control (PC) for anticancer effect and was used at the concentration of 2 μg/mL.

#### 3.2.5. Cell Metabolic Activity in MTT Reduction Assay

Cell metabolic activity was assessed by MTT reduction assay using gastric cancer AGS cells and reference L929 fibroblasts according to ISO 10993-5:2009. Cells were exposed to 5-FU, His, His-5-FU, PLA-OH, PLA-5-FU, PLA-His, and PLA-His-5-FU over the concentration range used for cytotoxicity screening. Dose–response curves were generated for all treatment groups. Apparent IC_50_ values were calculated from the MTT viability data. When the 50% viability threshold was crossed between two tested concentrations, IC_50_ was estimated by interpolation. When cell viability was already below 50% at the lowest concentration tested, IC_50_ was reported as <0.25 µg/mL. When cell viability remained above 50% within the tested concentration range, IC_50_ was reported as >2.5 µg/mL.

For qualitative uptake/cell imaging, AGS cells were exposed to the tested compounds at 0.5 µg/mL for 24 h. This concentration was selected to visualize cellular association/internalization while limiting excessive cytotoxic stress during microscopic observation. For all subsequent mechanistic analyses, the concentration of 1 µg/mL was selected based on the dose–response cytotoxicity profile in AGS cells, cytocompatibility assessment in reference L929 fibroblasts, and the need to preserve sufficient viable and analyzable cells for downstream assays. At 1 µg/mL, the tested PLA-based NPs fulfilled the ISO 10993-5 cytocompatibility criterion in L929 fibroblasts, while remaining biologically active against gastric cancer AGS cells. Therefore, 1 µg/mL was used for mitochondrial membrane potential analysis, TUNEL assay, cell cycle assessment, proliferation assay, lipid peroxidation analysis, ICAM-1 determination, cytokine secretion analysis, and NF-κB activation assay.

#### 3.2.6. Mitochondrial Membrane Potential

Human AGS cells were seeded at a density of 4 × 10^5^ cells/mL into a 96-well plate and cultured in a growth medium for 24 h at 37 °C and 5% CO_2_ to facilitate adhesion. Then, cells were exposed to various NPs, including PLA-OH, PLA-His, PLA-5-FU, and PLA-His-5-FU or 5-FU, His, or His-5-FU alone for 24 h, 48 h and 72 h. Mitochondrial membrane potential (MMP) was assessed according to the manufacturer’s procedure (Merck, Millipore, Burlington, MA, USA) by monitoring the fluorescence intensity (ex = 490, em = 525 nm) and that of the JC-10 dye (ex = 540, em = 590 nm) for ratio analysis. The ratio of red/green fluorescence intensity is used to determine MMP.

#### 3.2.7. Cell Cycle Assessment

Human AGS cells were seeded at a density of 1 × 10^6^ cells/mL into a 6-well plate and cultured in a growth medium for 24 h at 37 °C and 5% CO_2_ to facilitate adhesion. Then, cells were exposed to various NPs, including PLA-OH, PLA-His, and PLA-His-5-FU or 5-FU, His, or His-5-FU alone, for 24 h, 48 h, and 72 h. Cell cycle assessment was performed according to the manufacturer’s procedure (Thermo Fisher, Waltham, MA, USA). Cells were washed with PBS (Merck KGaA, Darmstadt, Germany), then treated with trypsin solution and centrifuged (10 min/300× *g*). The supernatant was removed, and the pellet was suspended in 500 µL of PBS and mixed thoroughly. Cells were then fixed by adding 70% cold ethanol to a final volume of 4500 µL and mixing continuously. Cell fixation continued at 4 °C for 24 h. The cell pellet was washed three times in PBS, and the cells were centrifuged each time (10 min/300× *g*). Cells were stained with propidium iodide (PI) solution for 30 min according to the manufacturer’s FxCycle PI/RNase Staining protocol. Cell cycle phases were assessed in a flow cytometer at an excitation wavelength of 488 nm, and emission wavelength was collected in the 564–606 nm range using a 585/42 bandpass filter. Each time, 10,000 cells were analyzed, and the percentage of cells in each phase of the cycle was determined. The cell cycle distribution was then analyzed in an LSR II Flow Cytometer (Becton Dickinson, Mountain View, CA, USA). The percentage of cells in G1, S and G2/M phases of the cell cycle was determined with the FlowJo 10.10.0 analytical software (flowjo.com).

#### 3.2.8. Confocal Microscopy

Confocal microscopy was performed to qualitatively assess cellular association/internalization of 5-FU/His-5-FU-containing NPs. The signal was detected based on the weak native blue fluorescence of 5-FU/His-5-FU. Samples were excited using the (405 nm) violet laser of the confocal microscope, and fluorescence emission was measured at 450 nm/430–480 nm. No external fluorescent dye was used to label either the drug or the NPs. Due to the low intrinsic fluorescence intensity of 5-FU, the obtained images were interpreted as qualitative evidence of cellular association/internalization rather than as quantitative measurements of intracellular drug accumulation [65,66]. Data analysis was performed using ImageJ software version 1.48v, with three independent repetitions conducted for each experimental variant.

#### 3.2.9. Preparation of Cells for Immunohistochemical Staining

Following the stimulation with NPs tested as described above, cell monolayers were rinsed three times with PBS. The cells were fixed in a 4% paraformaldehyde solution (Merck KGaA, Darmstadt, Germany) for 10 min at room temperature and washed as above. Next, cells were permeabilized using a 0.02% Triton X-100 solution (Merck KGaA, Darmstadt, Germany) for 10 min at room temperature, followed by washing with PBS. Any remaining gaps on the plate were blocked using 3% bovine serum albumin (BSA) (Merck KGaA, Darmstadt, Germany) in PBS for 1 h at room temperature. Finally, the cells were stained with the appropriate antibodies as outlined in the methods below.

#### 3.2.10. Lipid Peroxidation

Human AGS cells were exposed to various NPs, including PLA-OH, PLA-His, and PLA-5-FU or 5-FU, His, and His-5-FU. The cells were then immunohistochemically stained for 4-hydroxynonenal (4HNE) using rabbit anti-4HNE antibodies fluorescently labeled with Alexa Fluor 560 (Bioss, Woburn, MA, USA), as previously described [67]. Fluorescence intensity was measured using a multifunctional spectrophotometer, SpectraMax i3 (Molecular Devices, San Jose, CA, USA), at excitation and emission wavelengths of 550 nm and 590 nm, respectively. The results for 4HNE levels were presented in relative fluorescence units (RFUs). Three independent experiments were carried out in triplicate.

#### 3.2.11. Apoptosis

Human AGS cells, following exposure to NPs (PLA-OH, PLA-His, PLA-5-FU, and PLA-His-5-FU or 5-FU, His, or His-5-FU), were used for the development of terminal deoxynucleotidyl transferase dUTP nick end labeling (TUNEL) assay (Thermo Fisher Scientific, Waltham, MA, USA). The fluorescence intensity was measured with a multifunctional spectrophotometer, SpectraMax i3 (Molecular Devices, San Jose, CA, USA), at the following wavelengths: 488 nm (excitation) and 496 nm (emission) for assessing DNA damage, and 782 nm (excitation) and 805 nm (emission) for the TUNEL assay. TUNEL results were reported in RFUs. Three independent experiments were conducted in triplicate.

#### 3.2.12. Cell Proliferation

Cell proliferation was evaluated using the fluorescent CyQUANT Cell Proliferation Assay Kit (Invitrogen, Carlsbad, CA, USA) according to the manufacturer’s guidelines. Fluorescence was measured with the multifunctional spectrophotometer SpectraMax i3 (Molecular Devices, San Jose, CA, USA) at 782 nm (excitation) and 805 nm (emission). Each experimental variant was tested in triplicate.

#### 3.2.13. NF-kB Activation, Cytokine Production and ICAM-1 Deposition

THP-1 blue™ cells (Invitrogen, Carlsbad, CA, USA), derived from the human THP-1 monocyte line, containing NF-κB-responsive alkaline phosphatase (SEAP) reporter, were used to test whether tested NPs, including PLA-OH, PLA-His, PLA-5-FU, and PLA-His-5-FU or 5-FU, His, and His-5-FU alone could activate monocytes by inducing the NF-κB signaling pathway. SEAP was detected spectrophotometrically using Quanti-Blue (Invitrogen, Carlsbad, CA, USA). Cells were cultured at 37 °C with 5% CO_2_ in RPMI-1640 with 10% FBS, HEPES, 100 U/mL penicillin/streptomycin, 2 mM/mL glutamine, and 2 µg/mL blastidin (Thermo Fisher Scientific, Waltham, MA, USA). For activation assay, cells (4 × 10^5^ cells/mL) were stimulated with *Escherichia coli* NPs or LPS (O55:B5) (Sigma-Aldrich, Darmstadt, Germany) as a positive control (1 µg/mL) for 24 h, 48 h and 72 h. The culture medium consisted of a negative control. SEAP activity was measured at 650 nm using a plate reader. Each variant was tested in triplicate.

The level of transmembrane intracellular adhesion molecule (ICAM)-1 in gastric cancer cells was evaluated using immunofluorescence staining after cell exposure to compounds tested. The cells (1 × 10^6^ cells/mL) were grown in 96-well plates under standard conditions. Following a 15-min fixation with 4% formaldehyde, the wells were blocked for 2 h with a 3% BSA/PBS to prevent non-specific reagent binding. Primary antibodies against ICAM-1 (Santa Cruz Biotechnology, Dallas, TX, USA) were diluted in 1% BSA/PBS and added to cells, followed by incubation for 24 h at 37 °C, 5% CO_2_. Subsequently, the cells were treated with secondary Alexa Fluor 546-labeled antibodies (Invitrogen, Carlsbad, CA, USA), as previously described [68]. Fluorescence was measured using a multifunctional spectrophotometer SpectraMax i3 (Molecular Devices, San Jose, CA, USA), at an excitation wavelength of 556 nm and an emission wavelength of 573 nm. Each variant was tested in triplicate.

In parallel, supernatants were collected from AGS cell cultures after 24 h, 48 h and 72 h exposure to tested compounds as described above to assess the production of tumor necrosis factor alfa (TNF-α) and interleukin 1 beta (IL-β) by the Enzyme-Linked Immunosorbent Assay (ELISA) (sensitivity 1 pg/mL) according to the manufacturer’s procedure (Invitrogen, Carlsbad, CA, USA).

#### 3.2.14. Statistical Analysis

Statistical analyses and graph preparation were performed using GraphPad Prism 10.0 (GraphPad Software, Inc., San Diego, CA, USA; https://www.graphpad.com/). Statistical analysis was chosen based on data distribution and variance homogeneity. Data were analyzed using a parametric test, ANOVA with Games–Howell post hoc test (among the three or more tested variants) or unpaired Student’s *t*-test or Welch’s *t*-test (for pairwise comparison). When assumptions for parametric tests were not met, data were analyzed with a non-parametric Kruskal–Wallis test, followed by Dunn’s post hoc test (among the three or more tested variants) with 95% confidence (99% confidence intervals (1.00 − 0.05/5 = 0.99) or Mann–Whitney U test with a 95% confidence interval (for pairwise comparison). Data assumed to follow a normal distribution were examined using the Shapiro–Wilk test. The Brown–Forsythe test was used to test the equality of the group variances. Statistically significant results are indicated by a *p*-value of <0.05. The specific statistical tests used for each experiment are detailed in the figure legends.

## 4. Conclusions

The use of His-functionalized PLA NPs loaded with 5-FU represents a promising strategy to enhance the biological activity of 5-FU against gastric cancer cells. The present study showed that PLA-His-5-FU NPs exerted stronger anticancer effects against AGS cells *in vitro* than non-functionalized PLA-5-FU NPs or soluble 5-FU. PLA-His-5-FU induced apoptosis, reduced mitochondrial membrane potential, interfered with cell cycle progression, and decreased cell proliferation. In addition, PLA-His-5-FU promoted the secretion of pro-inflammatory cytokines, including TNF-α and IL-1β, and activated NF-κB signaling in monocytes, which suggests that this formulation may contribute to the development of an immune-activating microenvironment. Importantly, the tested NPs did not increase ICAM-1 deposition on AGS cells, which may be beneficial in limiting ICAM-1-dependent cancer cell spreading.

The present study demonstrates that His-functionalized PLA NPs loaded with 5-FU exert enhanced anticancer activity against gastric cancer AGS cells *in vitro* compared to non-functionalized PLA-5-FU NPs or soluble 5-FU. PLA-His-5-FU reduced mitochondrial membrane potential, promoted DNA fragmentation, interfered with cell cycle progression, and decreased cell proliferation. The formulation also affected selected immune-related readouts, including TNF-α and IL-1β secretion and NF-κB activation in THP-1 blue monocytes. These immune-related findings require confirmation under endotoxin-controlled conditions.

The observed effects should be interpreted as enhanced combined activity of His functionalization and 5-FU loading, rather than as evidence of pharmacological synergy. Further studies using combination index, isobologram, Bliss independence, or Loewe additivity models will be required to determine whether His and 5-FU interact synergistically in this NP-based system.

## Figures and Tables

**Table 1:** IC_50_ values for AGS cells of the tested compounds.

Tested Sample	IC_50_ 24 h (µg/mL)	IC_50_ 72 h (µg/mL)
PLA-OH	>2.5	>2.5
PLA-5-FU	~0.40	<0.25
PLA-His-5-FU	~0.25	<0.25
His	~1.74	~0.98
His-5-FU	~0.31	~1.07
5-FU	~0.84	~0.90

## Data Availability

This published article includes all data generated or analyzed during this study.

## Supplementary Materials

The following supporting information can be downloaded at: https://www.mdpi.com/article/10.3390/molecules31142520/s1.

## Abbreviations

The following abbreviations are used in this manuscript:

NPs	Nanoparticles
PLA	Polylactic acid
His	Histamine
5-FU	5-fluorouracil
TNF-α	Tumor necrosis factor alpha
NF-κB	Nuclear factor kappa B
THP-1	Human monocytic leukemia cell line
ICAM-1	Intercellular adhesion molecule 1
IARC	International Agency for Research on Cancer
GC	Gastric cancer
*H. pylori*	*Helicobacter pylori*
DDS	Novel drug delivery system
DOX	Doxorubicin
SEC	Size-exclusion chromatography
FTIR	Fourier-transform infrared spectroscopy
DGTS	Deuterated triglycine sulfate
ATR	Attenuated total reflectance
FBS	Fetal bovine serum
NC	Negative control
PC	Positive control
MTT	(3-(4,5-dimethylthiazol-2-yl)2,5-di-phenyltetrazolium bromide)
ISO	International Organization for Standardization
MMP	Mitochondrial membrane potential
PBS	Phosphate-buffered saline
PI	Propidium iodide
BSA	Bovine serum albumin
4HNE	4-hydroxynonenal
RFU	Relative fluorescence units
TUNEL	Terminal deoxynucleotidyl transferase dUTP nick end labeling
SEAP	Secreted alkaline phosphatase
HEPES	4-(2-hydroxyethyl)-1-piperazineethanesulfonic acid
IL-1β	Interleukin 1 beta
ELISA	Enzyme-Linked Immunosorbent Assay
ANOVA	Analysis of variance
MDR	Multi-drug resistance
NDDS	Nano-drug delivery systems
SEC	Solid Ehrlich carcinoma
SEM	Scanning Electron Microscopy
EE	Encapsulation efficiency
