# Supplementary material for: Mechanistic Insights into the Action of Histamine-Functionalized PLA Nanoparticles Loaded with 5-Fluorouracil Against Gastric Cancer Cells In Vitro
Source: Molecules. 2026 Jul 20;31(14):2520. doi: 10.3390/molecules31142520 (PMC13416300; doi:10.3390/molecules31142520)
Supplement: Supplementary file 1 [file molecules-31-02520-s001.zip › molecules-4398694-supplementary.pdf]

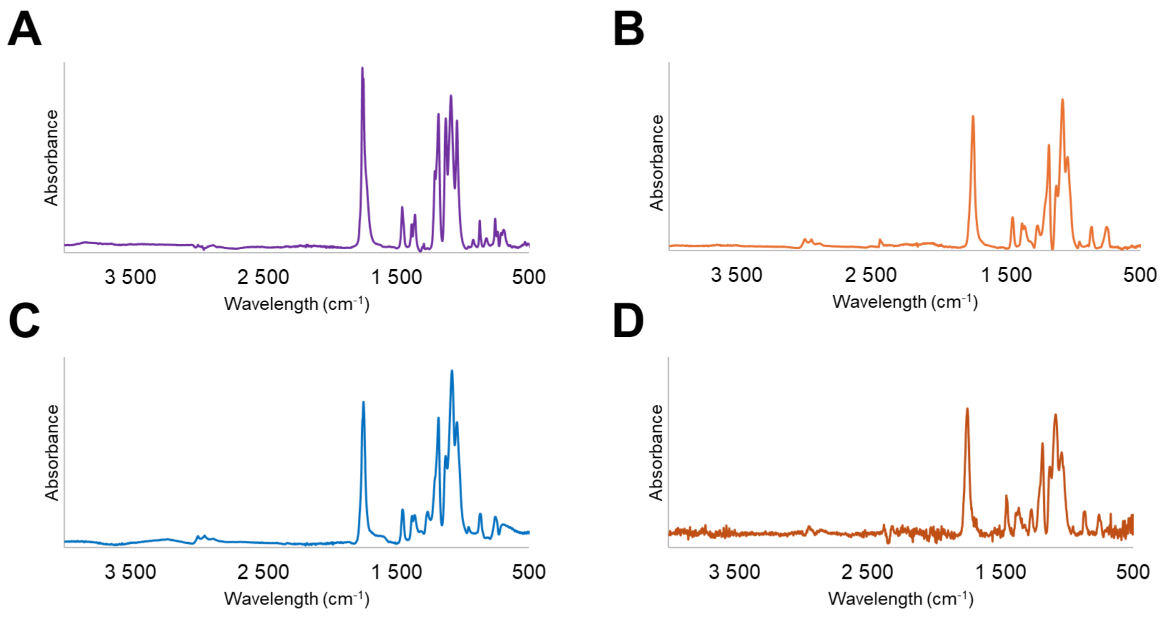

Figure S1. FTIR profiles of nanoparticles (NPs) composed of polylactic acid (PLA). (A) Empty NPs (PLA-OH), (B) PLA NPs functionalized with histamine (PLA-His), (C) PLA NPs loaded with 5-fluorouracil (PLA-5-FU), (D) PLA NPs modified with histamine and loaded with FU (PLA-His-5-FU).

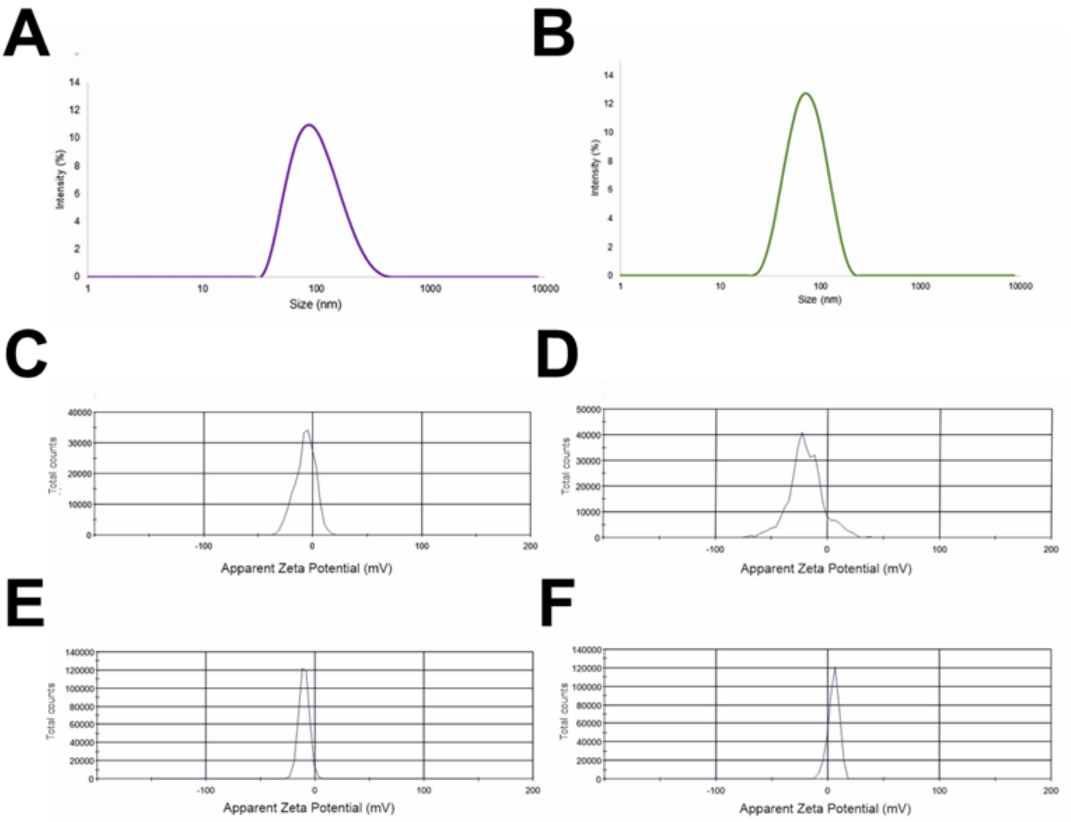

Figure S2. The size of nanoparticles (NPs) prepared by nanoprecipitation composed of polylactic acid (PLA), modified with histamine (His) and loaded with 5-fluorouracil (5-FU). (A) Size of NPs PLA-5-FU, (B) Size of NPs PLA-His-5-FU. Zeta potential graphs of: (C) empty NPs PLA-OH, (D) NPs PLA-His, (E) NPs PLA-5-FU, (D) NPs PLA-His-5-FU.

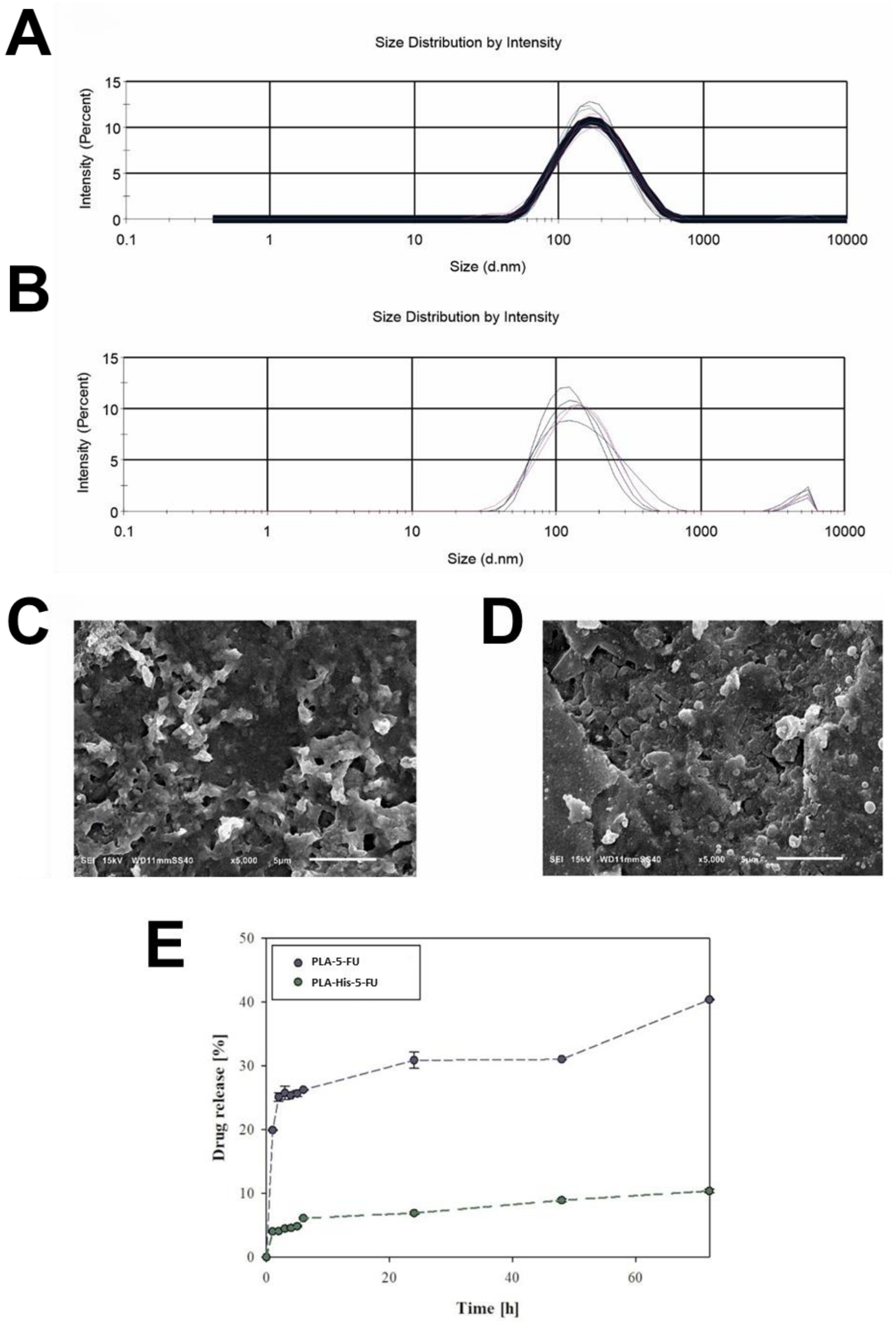

Figure S3. The stability and encapsulation effectiveness of PLA-composed NPs. The stability of nanoparticles (NPs) composed of polylactic acid (PLA) functionalized with histamine and loaded with 5-fluorouracil (PLA-His-5-FU) in PBS after 24 h (A and B). Scanning microscopy graphs of PLA NPs: (C) loaded with 5-FU (PLA-5-FU) or (D) modified with histamine and loaded with 5-FU (PLA-His-5-FU). The encapsulation efficiency (EE) of NPs PLA-5-FU and PLA-His-5-FU (E).

Table S1. Summary of the obtained nanoparticles (NPs).

NPs	Size[a] [nm]	PDI	Zeta potential[b] [mV]	EE[c] (%)
**PLA-OH**	**103**	0.21	-6.8	-
**PLA-His**	**147**	0.14	-18.5	-
**PLA-5-FU**	**121**	0.32	-10	34.8
**PLA-His-5-FU**	**150**	0.21	+5,2	34.6

[a] Determined by DLS at 25 °C, an average of three measurements from the intensity distribution curves.
[b] Determined by ZetaSizer.
[c] The amount of encapsulated 5 fluorouracil (5-FU) in nanoparticles was determined by UV–vis spectroscopy at 265 nm (for FU). EE - encapsulation efficiency. PLA-polylactic acid (PLA), empty PLA composed NPs (PLA-OH), NPs composed of PLA modified with histamine (PLA-His), PLA NPs loaded with FU (PLA-5-FU), NPs modified with histamine and loaded with FU (PLA-His-5-FU), PDI, Polydispersity Index.

Table S2. Drug release kinetics of the 5-fluorouracil (5-FU) loaded nanoparticles (NPs).

Sample	Kinetic model	R^2	k	N
PLA-5-FU	Korsemeyer - Peppas	**0.9614**	**20.3**	**0.13**
PLA-HIS-5-FU	Korsemeyer - Peppas	**0.9685**	**3.7**	**0.22**

PLA-5-FU-NPs composed of polylactic acid (PLA) and loaded with 5-FU; PLA-His-5-FU-NPs composed of PLA modified with histamine (His) and loaded with 5-FU. R^2, coefficient of determination; k, kinetic constant; n, release exponent.

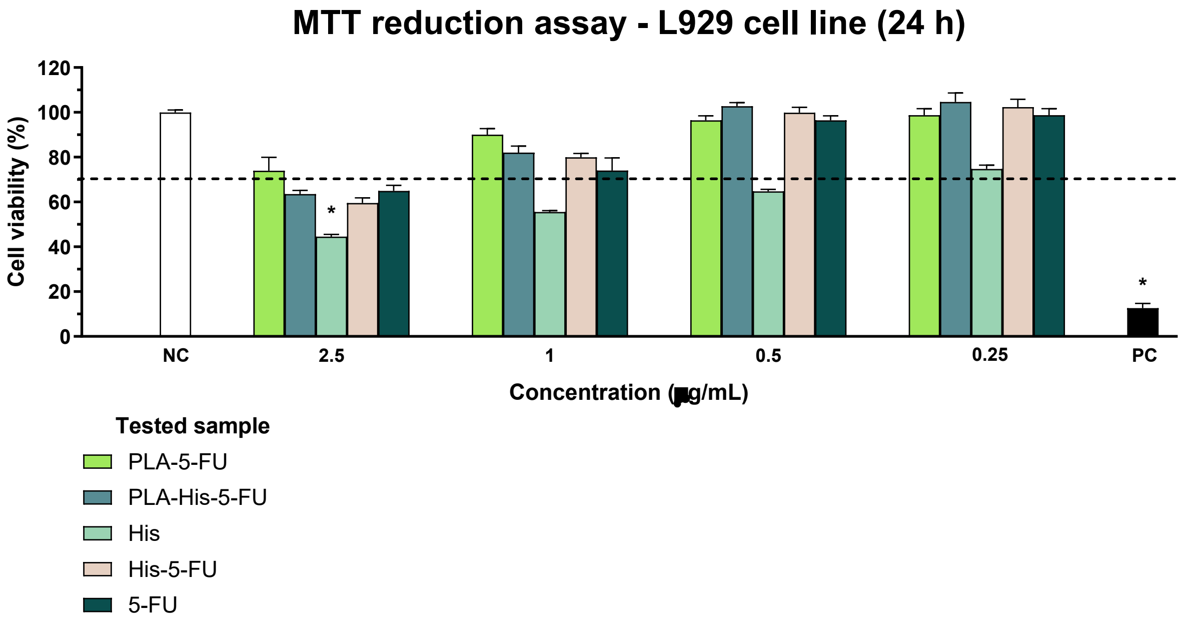

Figure S4. The cytotoxicity of PLA-composed nanoparticles on the reference mouse fibroblast L929. The biocompatibility of nanoparticles (NPs) composed of polylactic acid (PLA): empty NPs (PLA-OH), NPs loaded with 5-fluorouracil (PLA-5-FU), NPs modified with histamine (His) and loaded with FU (PLA-His-5-FU) and soluble components was assessed after 24 hours using the MTT [3-(4,5-dimethylthiazol-2-yl) 2,5-diphenyltetrazolium bromide] reduction assay, conducted according to ISO norm 10, 993-5 (Biological evaluation of medical devices – Part 5: Tests for *in vitro* cytotoxicity). * cells in medium vs cells exposed to studied components.

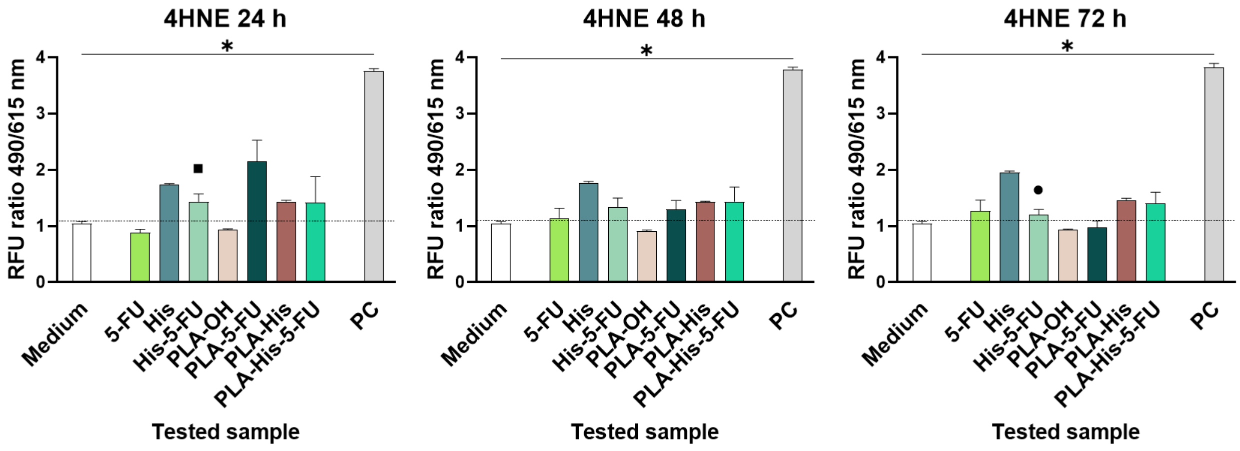

Figure 5. The 4-hydroxynonenal (4HNE) level in human gastric cancer cells AGS after stimulation with PLA-composed nanoparticles. The level of lipid peroxidation was measured after 24, 48 and 72 hours of cell exposure to nanoparticles (NPs) composed of polylactic acid (PLA): empty NPs (PLA-OH), NPs loaded with 5-fluorouracil (PLA-5-FU), NPs modified with histamine (His) and loaded with 5-FU (PLA-His-5-FU) or to soluble components (1 µg/mL). * cells in medium alone vs cells exposed to compounds tested, • His vs. His-5-FU or PLA-5-FU vs. PLA-His-5-FU.
